# Fortieth anniversary reflections on the early days of HIV and the current era

**DOI:** 10.1002/jia2.25757

**Published:** 2021-06-07

**Authors:** Kenneth H Mayer

**Affiliations:** ^1^ Fenway Health The Fenway Institute Boston MA USA; ^2^ Department of Medicine Beth Israel Deaconess Medical Center Boston MA USA; ^3^ Department of Medicine Harvard Medical School Boston MA USA

**Keywords:** HIV, AIDS, COVID‐19

In the summer of 1980, I began an Infectious Disease fellowship in Boston because of an increasing interest in understanding how microbes affected society. My laboratory projects focused on developing skills to track the molecular epidemiology of antibiotic resistance [1], but I also wanted to engage in community work and social medicine. I began working at Fenway Health, a health centre in Boston, committed to providing economically accessible and culturally appropriate care for sexual and gender minority patients. Prior to 5 June 1981, I had anticipated the worlds of academic research and community‐based sexually transmitted disease care to remain separate. So, it was with intense interest that I read the US CDC’s brief article in the *Morbidity and Mortality Weekly Report* (MMWR) about distinctive diseases appearing in “active homosexuals” in Los Angeles associated with immunosuppression [2]. This was followed a month later by another *MMWR* announcement of more cases appearing in San Francisco and New York City [3]. Other reports soon expanded the geographical range of the new syndrome, and noted a similar set of opportunistic infections and cancers among people who injected drugs.

The pandemic worsened quickly as more cases were identified, and in the absence of an identified aetiological agent, societal responses bifurcated. Because of the initial lack of US and global commitment to supporting an accelerated research programme or services for those in need, and as those most heavily impacted belonged to highly stigmatized populations, the responsibility for caring for the increasing number of desperately ill people was addressed by front‐line clinicians and newly constituted community‐based organizations that sprung up to provide meals, home care and other much needed assistance.

It took more than three years from that first report in the *MMWR* to the identification of HIV, the aetiological agent of AIDS, and another year before a commercially available diagnostic test became available. It took another 11 years before a meaningful treatment paradigm emerged, based on virological detection with newly developed nucleic amplification testing (NAAT) and the use of combination highly active antiretroviral therapy that targeted multiple steps in the virus life cycle. The timeline would invariably have been longer if community‐based activism had not arisen to address the neglect of global leaders and normative bodies. As more sexually and gender minority people felt increasingly vulnerable to an escalating pandemic, they repurposed lessons from the US Civil Rights and Women’s movements, as well as Gay Liberation to challenge researchers and public health officials to rethink traditional ways of conducting clinical trials and engaging at risk stakeholders [4].

The infusion of enhanced accountability in an era of increasingly instant communication led by community‐based groups like ACT‐UP influenced the conduct of subsequent research, including testing of vaccines against SARS‐CoV‐2. Moreover, activism that was initially US‐centric morphed into a multicentric international movement with indigenous leadership, like the Treatment Action Campaign. In an era of U = U and PrEP, the inevitability of powerful and successful tools may seem obvious, but decades of suffering, advocacy and careful clinical investigation led to these outcomes. And despite these advances, we remain without a safe and effective vaccine or cure, so the AIDS pandemic is far from over.

The 40th anniversary of the first report of AIDS, is an appropriate time to reflect on what we learned that can inform our efforts to stem to the global spread of SARS‐CoV‐2. The global HIV response has demonstrated that supporting basic and clinical research yields multiple dividends, including the ability to jumpstart responses to future health crisis. Tools and templates created to address HIV have been leveraged to respond to SARS‐CoV‐2 [5]. For example NAAT screening is a mainstay of SARS‐CoV‐2 diagnostics. Clinical trials networks established to conduct multicentre HIV efficacy have been quickly repurposed to assess promising interventions against SARS‐CoV‐2. COVID‐19 activists who challenge governments and manufacturers to waive intellectual property rights in order to speed vaccine access include founders of ACT‐UP and the HIV Treatment Action Campaign in conjunction with newer activists.

As someone whose personal and professional identity was forged in the crucible of AIDS in the 1980s, I have thought about Karl Marx’s aphorism that “*History repeats itself, first as tragedy, second as farce*,” [6] when musing about unscientific and insensitive responses of some leaders. At the same time, the mobilization of scientists and public health leaders to develop, test and distribute safe and effective vaccines with unprecedented speed has been heartening. The “race against time” with AIDS took 15 painful years to develop an effective treatment model. The rate‐limiting steps to an effective COVID‐19 pandemic response are primarily due to the insufficient resources where they are most needed, which also was often the case with the global AIDS response, until multilateral initiatives to increase their engagement were widely implemented.

There are also longer view lessons from the AIDS epidemic that need to be considered when addressing future pandemics. As humans diminish centres of biodiversity by heavy deforestation, contact with new species and therefore new pathogens, is inevitable [7]. Global travel can lead to rapid spread of new viruses to susceptible humans, increasing the risks for new pandemics. Simple steps, like fully funding a global molecular surveillance system, can provide timely alerts about new organisms with pandemic potential. The success of such a system will also be dependent on the willingness of national authorities to share data. Now is the time for the mobilization of resources to ensure that everyone has access to SARS‐CoV‐2 vaccines and state‐of‐the‐art treatment, as a basic human right. Addressing these immediate issues is an essential first step, but if we are to avoid endless pandemic cycles, we also need to attend to the more entrenched structural inequities, since poverty, crowding, poor nutrition and insufficient access to healthcare will potentiate the reach of any new pathogen. We need to recognize that we live in a global gene pool, and if we do not act with a profound realization of our shared humanity, more catastrophic situations are inevitable.

## Competing interests

The authors have declared no conflict of interest.

## Authors’ contributions

KHM conceptualized and wrote the editorial.

## Funding

None declared.
